# Head Down Deep Breathing for Cardioversion of Paroxysmal Supraventricular Tachycardia

**DOI:** 10.1155/2018/1387207

**Published:** 2018-09-23

**Authors:** Steven Hoon Chin Lim, Shieh Mei Lai, Kelvin Cheok Keng Wong

**Affiliations:** ^1^Accident and Emergency Department, Changi General Hospital, Singapore; ^2^Cardiology Department, Changi General Hospital, Singapore

## Abstract

The first-line recommended treatment for stable paroxysmal supraventricular tachycardia (PSVT) is the use of vagal maneuvers. Often the Valsalva maneuver is conducted. We describe two patients who converted to sinus rhythm without complications, using a head down deep breathing (HDDB) technique.

## 1. Introduction

Supraventricular tachycardia (SVT) is used to describe any abnormal narrow (<120 milliseconds) QRS complex tachycardia (>100 bpm) which starts and ends suddenly. It suggests the involvement of tissue from or above the bundle of His. The cause of paroxysmal SVT (PSVT) is frequently attributed to a re-entry mechanism. [1]

The first-line recommended emergency treatment for stable regular, narrow complex tachycardia secondary to SVT is the utilization of vagal maneuvers [2] such as the Valsalva maneuver (VM) [3] or the carotid sinus massage.

We present 2 cases of PSVT who converted to sinus rhythm by Trendelenburg positioning of the patient with deep breathing, “head down deep breathing” (HDDB).

## 2. Case Report

**Figure 1:**
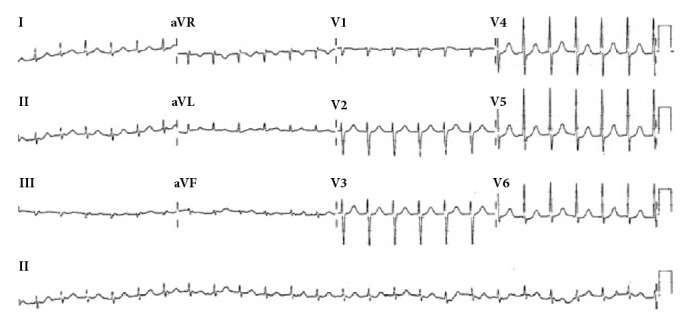
Patient A. Ventricular rate 149, SVT.

**Figure 2:**
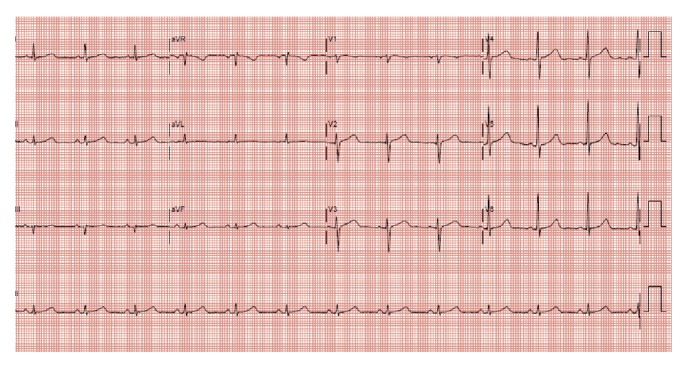
Patient A. Sinus rhythm, ventricular rate 75, and postconversion.

Patient A was a 65-year-old female with history of dyslipidemia, anemia, and postural hypotension with syncope. She experienced palpitations once every three months, each lasting about six hours which spontaneously resolved. She was referred from her General Practitioner's clinic for fever with upper respiratory tract symptoms for two days and a few hours of palpitations. There was no chest pain or shortness of breath. Her physical examination was largely unremarkable apart from regular tachycardia and blood pressure 166/61 mmHg. Her arrival electrocardiograph (ECG) revealed regular narrow complex tachycardia (see Figure 1). She was placed in Trendelenburg position and instructed to take in deep breaths and subsequently hold her breath for five seconds before exhalation. She did this and converted to sinus rhythm (see Figure 2) within five breaths. She tolerated the HDDB maneuver well. The immediate postmaneuver BP was 171/64 and 30 minutes later it was 121/65. She did not have any complications and was later discharged from the emergency department (ED).

**Figure 3:**
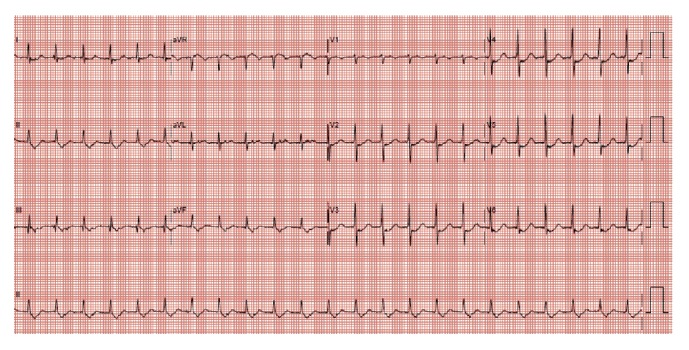
Patient B. 12-lead ECG, ventricular rate 138, and SVT.

Patient B was a 68-year-old female who had a history of hypertension and dyslipidemia. She felt sudden onset of palpitations associated with chest pain about 45 minutes before ED arrival. She had chest pain which radiated to her right shoulder and neck. She experienced some sweatiness but had no dyspnea or fever. At the ED, she was alert and not in pain or distress. On examination, she was tachycardic with normal blood pressure. Her lungs had clear air entry. Her ECG revealed SVT (see Figure 3). She was subjected to the similar head down deep breathing (HDDB) maneuver as described above and converted to sinus rhythm without any complications. Her postmaneuver BP was 131/73. Her initial serum Troponin T was 10 ng/L (normal lab range 0-29 ng/L). However, in view of the earlier presence of chest pain with cardiovascular risk factors, she was admitted to cardiology for observation. Her subsequent Troponin T levels remained normal, and an echocardiogram the next day showed normal left ventricular (LV) ejection fraction and diastolic function, with no structural heart disease. She was discharged thereafter.

## 3. Discussion

Vagal maneuvers are often used as first line treatment for haemodynamically stable SVT. By increasing vagal tone, there is slowing of conduction in the atrioventricular (AV) node, resulting in the termination of AV nodal dependent reentrant tachycardias like AVNRT and AVRT which constitutes the majority of regular narrow complex tachycardia.

Currently, the VM is likely the most commonly used vagal maneuver to treat stable PSVT. However, patient performance of the straining for VM is often inconsistent. Many patients may not be able to perform a persistent blow for 15 seconds and the blowing pressure is often not measured. The success rate is relatively low (5–20%) [4, 5]. Recently, a study by Appelboam et al. [3] which described a modified VM has a much higher success rate of 43%. The technique requires leg elevation and supine positioning of the patient at the end of the strain. Patients should not have orthopedic conditions which prevent them from assuming this body position.

Another vagal maneuver, the carotid sinus massage, is less commonly done and not favored due to invasiveness and the potential for injury to the carotid vessel. It is contraindicated in patients with murmurs or stenosis of the carotid arteries or history of transient ischemic attack/stroke due to the risk of neurological complications.

Hare and Ramlakhan have previously reported a child with recurrent SVT secondary to Wolff–Parkinson–White syndrome, consistently able to convert her SVT by doing a handstand [6]. They postulated that a handstand is likely to cause vagal stimulation by transiently increasing thoracic pressure, stimulating baroreceptor activity in the aortic arch and carotid bodies and resulting in increased parasympathetic tone. Their case demonstrated that although not widely recognized as a form of treatment, handstands can be a very effective and safe treatment. Similarly, Un et al. [7] have described sudden body posture changes to be effective. Here, the patients lie backward quickly from a seated to a supine position.

In another study, Waxman et al. described the capacity of deep inspiration and dependent body position to terminate tachycardia in 11 patients with recurrent PSVT. Their patients were referred for permanent atrial radio frequency pacemaker for self-termination of the arrhythmias. In eight of the patients, a deep inspiration and a dependent position repeatedly terminated episodes of PSVT [8].

It is believed that, during inspiration, pulmonary stretch receptors inhibit the efferent vagal tone. By deep breathing in a head down position, venous return to the heart is increased and contribute to a gradual elevation of blood pressure. At expiration, the removal of pulmonary stretch enhances efferent vagal tone. The vagal tone is also accentuated by baroreceptors due to raised blood pressure.

The clinical effectiveness and safety of HDDB for the termination of PSVT are unclear. We have described 2 patients who were successfully converted to sinus rhythm with HDDB as the primary treatment within a short time of presentation at A&E. Both patients recovered uneventfully without complications. In both cases, no VM (strain) nor sudden postural changes were necessary. It should be noted that both our patients are above 60 years old. However, there is insufficient information at this point to suggest that the technique is more effective in older patients or any particular age group. Further clinical studies are needed. Due to the dependency on transient elevation of blood pressure for HDDB to work, patients with markedly raised blood pressure may need to be excluded from this technique. Other considerations include patients with history of recent cardiac surgery or procedures, intracranial bleed, or vascular dissection; or the presence of underlying aortic, intracranial or other types of vascular aneurysm. The vagal tone is critical for the success of this technique so it may also be ineffective for patients on vagolytic drugs.

## 4. Conclusion

We describe 2 ED patients with PSVT who converted to sinus rhythm without complications using the HDDB technique. Further clinical studies are needed to compare the efficacy and safety of this simple technique against other vagal maneuvers like the Valsalva Maneuver for the treatment of stable PSVT. It may be a reasonable alternative technique.

## 5. EM Capsule

What do we already know about this clinical entity?

A dependent body position (handstand) and the conduct of deep breathing in a dependent body position have been shown to be effective at converting patients with SVT to sinus rhythm.

What is the major impact of this technique?

HDDB may be a simple alternative to VM (strain method) for the treatment of stable PSVT patients.

How might this improve emergency medicine practice?

Further studies are needed on the safety and efficacy of HDDB. This may be a simple, safe, and viable addition to the current forms of vagal maneuver.

## References

[1] Al-Zaiti S. S., Magdic K. S. (2016). Paroxysmal Supraventricular Tachycardia: Pathophysiology, Diagnosis, and Management. *Critical Care Nursing Clinics of North America*.

[2] Ching C. K., Leong S. H. B., Chua S. J. T. (2017). Advanced cardiac life support: 2016 Singapore guidelines. *Singapore Medical Journal*.

[3] Appelboam A., Reuben A., Mann C. (2015). Postural modification to the standard Valsalva manoeuvre for emergency treatment of supraventricular tachycardias (REVERT): A randomised controlled trial. *The Lancet*.

[4] Lim S. H., Anantharaman V., Teo W. S., Goh P. P., Tan A. T. H. (1998). Comparison of treatment of supraventricular tachycardia by Valsalva maneuver and carotid sinus massage. *Annals of Emergency Medicine*.

[5] Smith G., Morgans A., Boyle M. (2009). Use of the Valsalva manoeuvre in the prehospital setting: A review of the literature. *Emergency Medicine Journal*.

[6] Hare M., Ramlakhan S. (2015). Handstands: A treatment for supraventricular tachycardia?. *Archives of Disease in Childhood*.

[7] Un H., Dogan M., Uz O., Isilak Z., Uzun M. (2016). Novel vagal maneuver technique for termination of supraventricular tachycardias. *The American Journal of Emergency Medicine*.

[8] Waxman M. B., Bonet J. F., Finley J. P., Wald R. W. (1980). Effects of respiration and posture on paroxysmal supraventricular tachycardia. *Circulation*.

